# Optimizing Filament-Based TCP Scaffold Design for Osteoconduction and Bone Augmentation: Insights from In Vivo Rabbit Models

**DOI:** 10.3390/jfb15070174

**Published:** 2024-06-25

**Authors:** Julien Guerrero, Ekaterina Maevskaia, Chafik Ghayor, Indranil Bhattacharya, Franz E. Weber

**Affiliations:** 1 Center of Dental Medicine, Oral Biotechnology & Bioengineering, University of Zurich, 8032 Zurich, Switzerland; 2 Center for Surgical Research, University Hospital and University of Zurich, 8032 Zurich, Switzerland; 3 Center for Applied Biotechnology and Molecular Medicine (CABMM), University of Zurich, 8032 Zurich, Switzerland

**Keywords:** additive manufacturing, filament-based scaffolds, bone tissue engineering, microarchitecture, osteoconduction, tri-calcium phosphate, bone regeneration, vertical bone augmentation, bone substitute, ceramics

## Abstract

Additive manufacturing has emerged as a transformative tool in biomedical engineering, offering precise control over scaffold design for bone tissue engineering and regenerative medicine. While much attention has been focused on optimizing pore-based scaffold architectures, filament-based microarchitectures remain relatively understudied, despite the fact that the majority of 3D-printers generate filament-based structures. Here, we investigated the influence of filament characteristics on bone regeneration outcomes using a lithography-based additive manufacturing approach. Three distinct filament-based scaffolds (Fil050, Fil083, and Fil125) identical in macroporosity and transparency, crafted from tri-calcium phosphate (TCP) with varying filament thicknesses and distance, were evaluated in a rabbit model of bone augmentation and non-critical calvarial defect. Additionally, two scaffold types differing in filament directionality (Fil and FilG) were compared to elucidate optimal design parameters. Distance of bone ingrowth and percentage of regenerated area within scaffolds were measured by histomorphometric analysis. Our findings reveal filaments of 0.50 mm as the most effective filament-based scaffold, demonstrating superior bone ingrowth and bony regenerated area compared to larger size filament (i.e., 0.83 mm and 1.25 mm scaffolds). Optimized directionality of filaments can overcome the reduced performance of larger filaments. This study advances our understanding of microarchitecture’s role in bone tissue engineering and holds significant implications for clinical practice, paving the way for the development of highly tailored, patient-specific bone substitutes with enhanced efficacy.

## 1. Introduction

In recent years, additive manufacturing has emerged as a revolutionary paradigm shift in the realm of biomedical engineering, particularly in the field of bone tissue engineering and regenerative medicine [1,2,3,4]. Unlike conventional subtractive methodologies, which entail carving away material from a bulk source, additive manufacturing builds objects layer-by-layer, affording unparalleled control over intricate designs and microarchitectures [5,6]. This capability holds immense promise for the fabrication of personalized bone substitutes, where precise macroarchitecture matching is paramount to ensuring optimal integration with the patient’s anatomy [7,8,9,10,11].

At the forefront of this technological advancement lies the concept of microarchitecture, a defining characteristic that governs the distribution of material within the overall macrostructure of a bone substitute [5]. While the macroarchitecture dictates the general shape and fit of the implant, the microarchitecture delves into the finer details, influencing crucial properties such as porosity and pore size [12]. These parameters, in turn, play a pivotal role in modulating osteoconduction, the process through which a scaffold guides the ingrowth of new bone tissue to bridge a defect [7,13,14].

Historically, the pursuit of ideal bone substitute microarchitectures has been shaped by evolving paradigms. Early studies advocated for smaller pore diameters based on observations of cancellous bone porosity, the gold standard for bone substitutes in clinical practice [15]. However, the advent of additive manufacturing has ushered in a paradigm shift, with recent investigations highlighting the benefits of larger pore sizes, ranging from 0.8 to 1.2 mm, for enhanced osteoconduction and bone regeneration [16,17].

While much attention has been devoted to the optimization of pore-based scaffold architectures, the exploration of filament-based microarchitectures remains relatively nascent [7,18]. Extrusion-based additive manufacturing methods, favored for their cost-effectiveness, have traditionally dominated scaffold production but are inherently limited in microarchitecture variability due to constraints imposed by filament dimensions and mechanics [19]. To address this limitation, our study employs a novel lithography-based additive manufacturing approach to mimic filament-based microarchitectures, offering a custom library of finely tuned filament-based scaffolds with unprecedented control over filament size, spacing, and directionality.

Engaging in a meticulous exploration, we examined three distinct filament-based scaffolds fabricated from tri-calcium phosphate (TCP) with filament thicknesses of 0.5 mm, 0.83 mm, and 1.25 mm. Our primary objective was to dissect the intricate interplay between filament characteristics and biological responses (i.e., bone ingrowth and neo-formation) using two in vivo rabbit models. Specifically, we employed both a bone augmentation model and a one-sided ingrowth model in cranial defects to conduct a comparative analysis. This approach enabled us to determine whether the optimal filament-based microarchitecture for promoting osteoconduction coincides with that for bone augmentation.

Moreover, in our quest to elucidate optimal design parameters for enhancing bone regeneration outcomes, we conducted a comparative analysis of two scaffold types differing in filament directionality (i.e., Fil- and FilG-related scaffolds). The Fil scaffolds feature 100% filament alignment with the advancement of bone in defects and osteoconduction and 0% alignment with bone augmentation. FilG scaffolds exhibit a 50% alignment with bone advancement in defects and bone augmentation due to 50% orthogonally oriented filaments. Through this comparative investigation, we aimed to uncover the most effective design parameters for guiding bone regeneration, thus advancing our understanding of filament-based scaffold design optimization.

This comprehensive analysis not only advances our fundamental understanding of microarchitecture’s role in bone tissue engineering but also holds profound implications for clinical practice [5]. By deciphering the intricate nuances of filament-based scaffold design, our study paves the way for the development of highly tailored, patient-specific bone substitutes with enhanced efficacy and clinical translatability.

## 2. Materials and Methods

### 2.1. Scaffold Production

**Figure 1:**
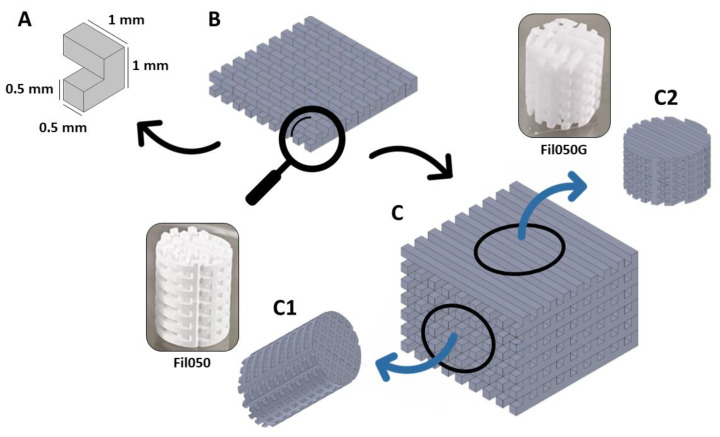
Construction of scaffold from the unit cell to microarchitecture. (**A**) A unit cell exemplified for filaments of 0.5 mm is patterned (**B**) and stacked (**C**) to form the filament-based microarchitecture. (**C**) Strategy to generate scaffolds with 100% and 50% directionality from the same microarchitecture. (**C1**) Fil050 and (**C2**) FilG050 are derived from the same microarchitecture.

The scaffolds were fabricated using unit cells composed of cubes. The measurements of cubes were 1.00, 1.75, or 2.50 mm in length (Figure 1A), resulting in three groups in total. Those cubes were then patterned (Figure 1B) and stacked (Figure 1C), enabling the construction of filament-based scaffolds designed to mimic filaments measuring 0.50 (Fil050), 0.83 (Fil083), or 1.25 (Fil125) mm in square (Figure 1A–C), identical in macroporosity (50%) and transparency (25%). Each group consisted of six scaffolds. TCP scaffolds were manufactured using TCP slurry LithaBone™ TCP 300 (Lithoz, Vienna, Austria) with a CeraFab 7500 system (Lithoz, Vienna, Austria) [20,21]. Each scaffold was built up layer-by-layer, with each layer of slurry (25 μm) solidified by exposure to blue LED light at a resolution of 50 μm along the x/y-plane. Following fabrication, the green body (i.e., printed scaffold before thermal decomposition of the binder and sintering of the ceramic particles) was carefully detached from the printer’s platform using a razor blade, then meticulously cleaned with LithaSol 20™ (Lithoz, Vienna, Austria) and dried with pressurized air. Subsequently, the polymeric binder was thermally decomposed, and the remaining ceramic particles were sintered to enhance density, with a dwell time of 3 h at 1100 °C. The resulting sintered TCP scaffolds were then transferred to a sterile environment, packaged for integration into the operational workflow, and utilized as implants for non-critical calvaria defects in rabbits without further sterilization.

### 2.2. Scaffold Implantation

All animal procedures were conducted in accordance with the guidelines set forth by the Animal Ethics Committee of the local authorities (Canton Zurich, 065/2018 and 090/2021) and adhered to the ethical standards outlined in the bylaws of the Institutional Animal Care and Use Committee [5]. Additionally, to prevent the scaffold placement on the calvaria from influencing the outcome, the arrangement pattern was rotated clockwise for each subsequent animal.

#### 2.2.1. Osteoconduction

For the in vivo model of osteoconduction, animal weights ranged from 3.5 to 4.5 kg, and a standard laboratory diet was provided throughout the study. Anesthesia was induced pre-surgery with ketamine (65 mg/kg) and xylazine (4 mg/kg) and maintained during the operation with a mix of isoflurane and O_2_. The procedure, as previously detailed, involved the insertion of scaffolds into calvarial defects of eight female New Zealand white rabbits aged 26 weeks [21]. Each animal received three filament-based scaffolds, resulting in a total of 24 implants. The scaffolds, measuring 7.5 mm for the 2.5 mm of the upper part and 6.0 mm in diameter for the 2.5 mm of the lower part. This restricted the advancement of the scaffold to the minimum calvarial bone thickness of 2.5 mm. The surgical procedure involved disinfection of the cranium, incision, soft tissue deflection, removal of periosteum, marking and completion of defects using trephine burrs, saline flushing of defects, and gentle press-fitting of implants, as previously shown [5]. The wounds were closed with sutures following implantation. Treatment modalities were randomized and allocated to animals, with conditions labeled as Fil050, Fil083, Fil125, and their “FilG” respective counterpart. Four weeks post-implantation, the animals were anesthetized and euthanized with an overdose of pentobarbital.

#### 2.2.2. Bone Augmentation

The scaffolds utilized for bone augmentation assessment exhibited a diameter of 6.00 mm and a height of 12.00 mm. The in vivo bone augmentation model has been previously detailed and illustrated [22]. Briefly, the procedure involved creating three evenly distributed circular slits with a diameter of 6.00 mm and a sink depth of 1.00 mm using a trephine. Subsequently, the external cortical plate within the circles was perforated three times using a 1.00 mm burr. Following irrigation of the surgical site, three titanium cylinders (7.00 mm in height and 7.00 mm in outer diameter) were securely fastened into the prepared slits to ensure primary stability. These cylinders were then filled with the respective filament-based scaffolds. Prior to insertion into the cylinders, the scaffolds were trimmed to a length of 5.00 mm using a scalpel, capped with titanium lids, and the skin was sutured to cover both the calvarial bone and the cylinders. Four weeks post-operation, the rabbits underwent general anesthesia and were euthanized with an overdose of pentobarbital. The entire cranium, encompassing all three cylinders, was excised and subsequently embedded for further analysis.

### 2.3. Histomorphometry

Following excision, the calvaria containing the scaffolds were embedded in methylmethacrylate (MMA) and sectioned into three pieces, each containing either an implant or a cylinder. Ground sections were then prepared from the middle of each implant or cylinder and stained with Toluidine-blue to facilitate visualization of neo bone tissue formation. Subsequently, these sections were photographed using image analysis software (Image-Pro Plus^®^; V11, Media Cybernetic, Silver Springs, MD, USA).

Concerning the quantitative analysis, in the bone augmentation model, the extent of bone ingrowth was determined by measuring the maximal stretch (i.e., distance in mm, illustrated in the Figures by a red dashed line), where the neo-formed bone was detected from the lower end of the titanium cylinder towards the lid. This measurement was represented as “vertical bone ingrowth”. Regarding the osteoconduction model, the extent of bone ingrowth was measured as the distance between each side of the bone defect margins and the distance where the neo-formed bone was detected in the center of the implanted scaffold. In this case, the measurement was represented as “1-sided bone ingrowth”. Additionally, the area of bony regeneration was quantified as the percentage of bone and bone-integrated scaffold within the cylinder for bone augmentation, and as half the area between the defect margins and the 2.50 mm restricted sink depth of the scaffolds for osteoconduction. Additionally, the area of bony regeneration was quantified as the percentage of bone and bone-integrated scaffold within the cylinder for bone augmentation, and as half the area between the defect margins and the 2.50 mm restricted sink depth of the scaffolds for osteoconduction.

### 2.4. Statistical Analysis

To evaluate osteoconduction within the calvarial defect, bone front ingrowth was assessed from both the left and right defect margins in the ground sections, with each half of the defect and its corresponding cylinder serving as the primary analysis unit. Data from 8 to 9 different rabbits were aggregated in each group, resulting in 8–18 samples per group. Prior to statistical analysis, normality of the datasets was assessed using the Shapiro–Wilk test. For datasets demonstrating non-normal distribution, the Kruskal–Wallis test for multiple comparisons and Dunn’s post-hoc test, or the Mann–Whitney test for single comparison, were employed. Conversely, datasets satisfying the normality test were analyzed using 1-way ANOVA with Bonferroni’s or Dunnet’s post-test for multiple comparisons, or t-test for single comparison. Additionally, the Jonckheere–Terpstra trend test [23] was used to evaluate for correlation between the variation in filament thickness and length of bone ingrowth or bone area regenerated in study samples. Statistical significance was defined as *p* values < 0.05 (* *p* < 0.05, ** *p* < 0.01, *** *p* < 0.001, and **** *p* < 0.0001), with corresponding values illustrated in graphs and data presented as either mean ± standard deviation or median ± lower/upper quartile. GraphPad Prism 9 Software (GraphPad Software Inc., San Diego, CA, USA) was utilized for data processing.

## 3. Results

### 3.1. Implantation of TCP-Based Scaffolds with TPMS Microarchitecture

All animals utilized in both in vivo models of bone augmentation or osteoconduction maintained robust health throughout the study period. The absence of inflammation and the presence of bone formation within the cylinders or at the defect site underscore the favorable biocompatibility of the TCP filament-based material and its designs. These findings affirm the suitability of the material for orthopedic and maxillofacial applications, emphasizing its compatibility with biological systems and potential for promoting bone healing without adverse reactions.

#### 3.1.1. Performance of Filament-Based Microarchitectures in Bone Augmentation

**Figure 2:**
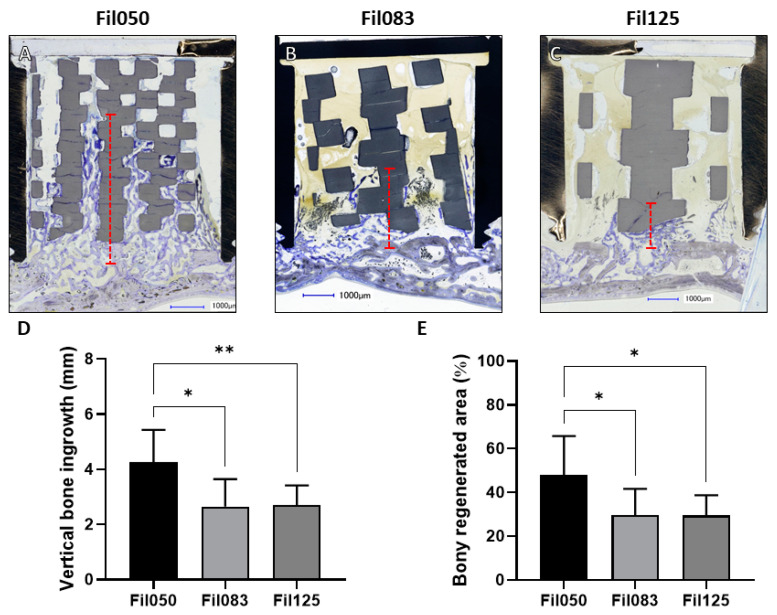
Vertical bone augmentation with respect to Fil-related scaffolds. (**A**–**C**) Central histological sections of cylinders extracted from rabbit crania after 4 weeks depicting (**A**) Fil050, (**B**) Fil083, and (**C**) Fil125 filament-based scaffolds. The distance from the top part of the calvaria to the highest point of bone ingrowth within the scaffold is illustrated by the red dashed line. In the histological sections, titanium cylinders appear black, scaffolds appear greyish, and bone appears greyish purple. Scale bars of 1.00 mm and filament thickness are provided. (**D**) Vertical bone ingrowth and (**E**) extent of bony regenerated area achieved for Fil050, Fil083, and Fil125 filament-based scaffolds. Values are presented as mean with standard deviation. Statistical significance is denoted by *p* values < 0.05 (* *p* < 0.05 and ** *p* < 0.01).

In all three filament-based microarchitectures implanted, there was evident bone tissue growth beyond the original calvarial bone boundary, as depicted in the stained histological sections (Figure 2A–C). This phenomenon underscores the promising potential of these scaffolds in facilitating bone regeneration. Specifically, within Fil050 constructs, bone tissue extended by an average of 4.27 ± 1.15 mm into the cylindrical structures, indicating robust bone augmentation properties. Conversely, for Fil083 and Fil125, the extent of advancement was significantly lower compared to Fil050 scaffolds, measuring at 2.64 ± 0.99 mm and 2.70 ± 0.70 mm, respectively (Figure 2D).

Quantitatively, the percentage area of bony regeneration provides further insight into the efficacy of these scaffold designs. For Fil050, the substantial value of 47.87 ± 17.87% highlights the significant contribution of this particular architecture to bone tissue formation. In contrast, both Fil083 and Fil125 exhibited significantly lower percentages, with 29.62 ± 11.97% and 29.44 ± 9.27%, respectively (Figure 2E). This disparity underscores the influence of scaffold microarchitecture on the extent of bone regeneration, with Fil050 demonstrating superior performance in this regard.

**Figure 3:**
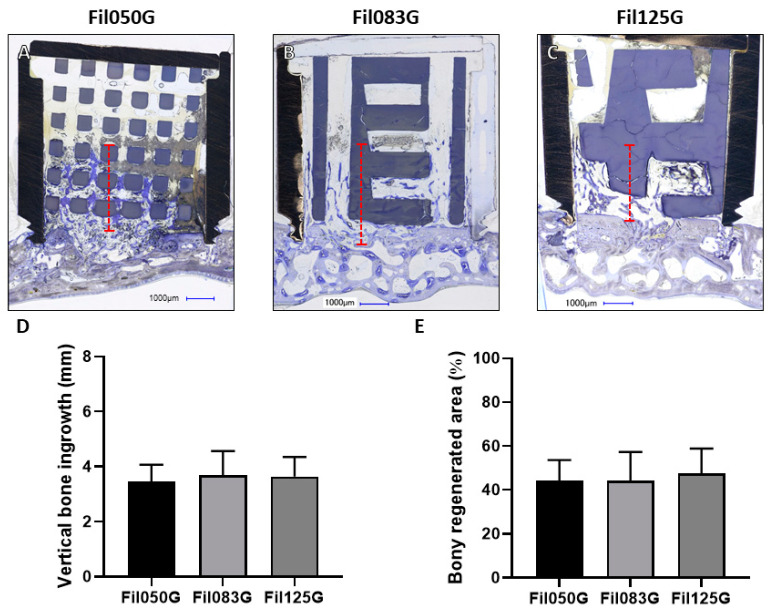
Vertical bone augmentation with respect to FilG-related scaffolds. (**A**–**C**) Central histological sections of cylinders extracted from rabbit crania after 4 weeks depicting (**A**) Fil050G, (**B**) Fil083G, and (**C**) Fil125G filament-based scaffolds. The distance from the top part of the calvaria to the highest point of bone ingrowth within the scaffold is illustrated by the red dashed line. In the histological sections, titanium cylinders appear black, scaffolds appear greyish, and bone appears greyish purple. Scale bars of 1.00 mm and filament thickness are provided. (**D**) Vertical bone ingrowth and (**E**) extent of bony regenerated area achieved for Fil050G, Fil083G, and Fil125G filament-based scaffolds. Values are presented as mean with standard deviation.

Regarding the corresponding “FilG” scaffolds, a similar trend of bone tissue progression was observed following staining of the histological sections (Figure 3A–C). However, no significant differences were observed between the three groups of scaffolds, indicating comparable osteogenic potential. Specifically, for Fil050G, bone tissue extended by an average of 3.45 ± 0.61 mm into the cylindrical structures, representing a consistent pattern of bone ingrowth. Similarly, Fil083G and Fil125G demonstrated advancements of 3.68 ± 0.88 mm and 2.63 ± 0.71 mm, respectively (Figure 3D).

The measurement of the percentage area of bony regeneration yielded similar results to the vertical bone ingrowth, further corroborating the findings. It was determined to be 44.22 ± 9.36% for Fil050G, 44.22 ± 13.03% for Fil083G, and 47.50 ± 11.36% for Fil125G (Figure 3E). This consistency underscores the reliability of the observed trends across different scaffold designs and suggests the robustness of the experimental outcomes.

#### 3.1.2. Performance of Filament-Based Microarchitectures in Osteoconduction

Scaffolds with identical filament-based microarchitectures (i.e., Fil and FilG), employed to evaluate vertical bone ingrowth in the in vivo model of bone augmentation, were also utilized to assess one-sided bone ingrowth in the in vivo model of non-critical calvarial defects as a metric of osteoconductivity.

**Figure 4:**
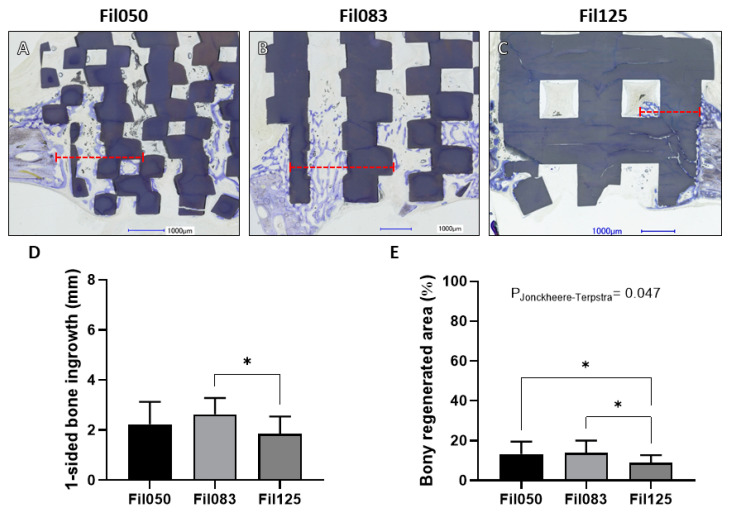
One-sided bone ingrowth regarding Fil-related scaffolds. (**A**–**C**) Central histologic sections of one-half of the 6.00 mm defects harvested from the crania of rabbits after 4 weeks are displayed depicting (**A**) Fil050, (**B**) Fil083, and (**C**) Fil125 filament-based scaffolds. The distance from the outer ring of the calvarial defect to the furthest point of bone ingrowth within scaffold is illustrated by the red dashed line. In the histological sections, scaffolds appear greyish, and bone appears greyish purple. Scale bars of 1.00 mm and filament thickness are provided. (**D**) One-sided bone ingrowth into the 6.00 mm defect and (**E**) percentage area of bony regeneration achieved for Fil050, Fil083, and Fil125 filament-based scaffolds. Values are presented as mean with standard deviation. Statistical significance is denoted by *p* values < 0.05 (* *p* < 0.05). The *p* values from the Jonckheere–Terpstra trend test are also provided.

Within the scaffold group featuring the “Fil” design (Figure 4A–C), the maximal bone ingrowth from one side into the defect, guided by the Fil050 microarchitecture, measured 2.23 ± 0.89 mm (Figure 4D). For Fil083, this figure increased to 2.62 ± 0.65 mm, significantly surpassing scaffolds with the Fil125 microarchitecture, where bone ingrowth reached 1.84 ± 0.70 mm (Figure 4D).

The percentage of bony regenerated area achieved by the different scaffolds within this group revealed that Fil050 reached 13.28 ± 6.28%, while Fil083 reached 13.89 ± 6.13%. Both percentages were significantly higher than that of Fil125, which reached 8.75 ± 3.93% (Figure 4E). Furthermore, a significant decrease in the percentage of bony regenerated area correlated with the increase in filament size between Fil050, Fil083, and Fil125 (*p*-value = 0.0047) was measured, as determined by the Jonckheere–Terpstra trend test (Figure 4E).

**Figure 5:**
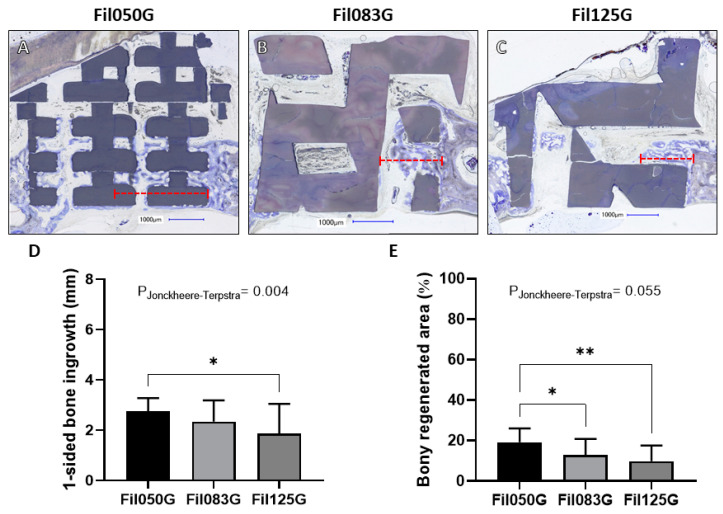
One-sided bone ingrowth regarding FilG-related scaffolds. (**A**–**C**) Central histologic sections of one-half of the 6.00 mm defects harvested from the crania of rabbits after 4 weeks are displayed depicting (**A**) Fil050G, (**B**) Fil083G, and (**C**) Fil125G filament-based scaffolds. The distance from the outer ring of the calvarial defect to the furthest point of bone ingrowth within scaffold is illustrated by the red dashed line. In the histological sections, scaffolds appear greyish, and bone appears greyish purple. Scale bars of 1.00 mm and filament thickness are provided. (**D**) One-sided bone ingrowth into the 6.00 mm defect and (**E**) percentage area of bony regeneration achieved for Fil050, Fil083, and Fil125 filament-based scaffolds. Values are presented as mean with standard deviation. Statistical significance is denoted by *p* values < 0.05 (* *p* < 0.05, and ** *p* < 0.01). The *p* values from the Jonckheere–Terpstra trend test are also provided.

In the group featuring the “FilG” design, as shown in the histological sections (Figure 5A–C), maximal bone ingrowth from one side into the defect, guided by the Fil050G microarchitecture, measured 2.75 ± 0.52 mm (Figure 5D). This was significantly higher than Fil083 with 2.34 ± 0.84 mm and scaffolds with Fil125G microarchitecture with 1.85 ± 1.19 mm (Figure 5D). Additionally, a significant decrease in the distance of bone ingrowth correlated with the increase in filament size between Fil050G, Fil083G, and Fil125G (*p*-value = 0.004) was also calculated, as demonstrated by the Jonckheere–Terpstra trend test (Figure 5D).

Regarding the percentage of bony regenerated area within this group, Fil050G achieved 19.22 ± 6.78%, significantly higher than Fil083G, which reached 12.84 ± 7.92%. Both were notably higher than Fil125G, which reached 9.82 ± 7.72% (Figure 5E). Furthermore, a significant decrease in the percentage of bony regenerated area correlated with the increase in filament size between Fil050, Fil083, and Fil125 (*p*-value = 0.0055) was observed, as determined by the Jonckheere–Terpstra trend test (Figure 5E).

## 4. Discussion

The integration of additive manufacturing into the realm of biomedical engineering represents a pivotal advancement, revolutionizing the landscape of bone tissue engineering and regenerative medicine [24]. Filament-based or extrusion-based microarchitectures are widely used due to the availability of low-cost machines and materials. The application of extrusion-based additive manufacturing, however, limits microarchitecture variability due to constraints imposed by filament dimensions and mechanics [25]. Traditionally, the optimization of bone substitute microarchitectures has predominantly centered on pore-based scaffold designs [26]; however, filament-based microarchitectures have received comparatively less attention [20]. Addressing this limitation, our study adopts a lithography-based approach, offering unprecedented control over filament characteristics such as size, spacing, and directionality [27] to produce a library of six filament designs. All these designs were identical in material, amount of material, macroporosity, and transparency. In terms of directionality of the filaments, the filaments of the Fil designs were oriented 100% along the x/y plane, whereas in the FilG designs, 50% were oriented along the x plane and 50% along the z plane.

In this study, we utilized female rabbits as our model organism. The selection was based on their physiological characteristics (female rather than male), which offer consistency and comparability with previous research in the field of bone formation [28]. Moreover, females can more easily be kept in groups. The choice of female rabbits also helps in maintaining a stable and predictable growth pattern, thereby minimizing variability in our experimental results. However, the impact of hormonal factors on bone formation could be another research direction for future experiments to further elucidate the complex interactions involved [29]. Rabbits provide a relevant model for studying bone repair due to their similarities to human bone healing processes [30]. However, it is important to acknowledge that rabbits, like all animal models, have inherent biological differences from humans, including variations in physiology, metabolism, and immune responses which can affect the translatability of the findings and are limitations of our study [31].

For bone augmentation, we found the Fil050 design to induce a superior bone ingrowth advancement and increased bony regenerated area (Figure 2) compared to all other designs, despite the fact that none of the filaments were oriented towards the direction of bone augmentation. For filament dimensions and distances exceeding 0.50 mm (Fil083, Fil125), however, the lack of filaments oriented towards the vertical bone ingrowth hampered bone ingrowth and the extent of bony regenerated area significantly. The alignment of 50% of the filaments with the vertical bone ingrowth direction, as realized with the FilG designs, was sufficient to yield good advancement of the bone front and bony regenerated area, irrespective of the dimension and distance of the filaments (Fil050G, Fil083G, Fil125G). Thus, filament direction towards the bone ingrowth direction is important for the extent of bone advancement and bone regeneration but can be overcome by small filaments (Fil050). Reports on microarchitecture and vertical bone augmentation are scarce. A single 3D-printed rod-based scaffold was generated by a digital light processing (DLP)-type 3D printer in HA/TCP and was shown in a dog defect augmentation model to perform significantly better than with particles [32,33]. A similar result was seen for a 3D-printed commercially available filament microarchitecture in comparison to particles (BioOss^®^ or Ceros^®^) [34]. Both results suggest that ordered rod- or filament-based 3D-printed microarchitectures perform better than random particle-based microarchitectures. The influence of directionality in vertical bone augmentation was tested with designs allowing cell migration, vessel formation, and bone formation in either 1D, 2D, or 3D [35,36]. Here, the restriction to 1D yielded increased and more advanced bone formation, supporting our finding that filament directionality has a positive effect on bone formation and extent of augmentation.

Since bone augmentation is a single directed bone ingrowth, we applied the same measure for cranial defects, where bone ingrowth occurs from any defect margin. One-sided bone ingrowth in the defect and bony regenerated area was highest with Fil050G and decreased in the FilG with increasing filament dimension and distance (Figure 5). Thus, if the filament direction is just 50% aligned to bone ingrowth, thin filaments (Fil050G) can compensate for the lower level of alignment. In the Fil series, with 100% alignment, Fil050 and Fil083 showed no difference, but the latter one induced a significant increase in bone ingrowth compared to Fil125. For bony regenerated area, increase of filament dimension and distance correlated with a decrease in bony regenerated area. In essence, the optimal filament design differs between bone augmentation and defect bridging. For defect bridging, the Fil050G design, and for bone augmentation the Fil050 design, is optimal. This discrepancy was also evident with pore-based microarchitectures since pores of 1.20 mm were ideal for defect bridging, but pores of 1.70 mm perform better for bone augmentation [37]. In both cases, the alignment with the bone growth direction was only 50 or 0% and shows that thin filaments at a low distance can compensate for lack of alignment with bone growth. Our comprehensive analysis not only advances our fundamental understanding of microarchitecture’s role in bone tissue engineering but also holds profound implications for clinical practice [38]. By deciphering the optimal design parameters in terms of filament thickness and directionality, our study contributes to the development of next-generation bone substitutes with enhanced biocompatibility and efficacy, ultimately benefiting patients in need of bone regeneration therapies [39].

Furthermore, our research sheds light on the importance of a uniform channel size, with 0.50 mm between filaments and 0.83 mm in the diagonal demonstrating optimal performance for bone augmentation, as well as being preferable for bone defect repair in accordance with others [40,41]. When we tested pore-based architectures for osteoconduction and bone augmentation [37], optimal pore size for osteoconduction was 0.80–1.20 mm and 1.70 mm for bone augmentation and therefore quite different. Pores 0.50 mm in diameter always performed the worst. If the microarchitecture changes from pore to channel-like designs, the optimal channel width for both models is 0.50 mm, pointing to a guiding effect of the filaments that is lacking in the pore-based designs. This is further supported by an overall superior performance of filaments, with a bone augmentation of 4.27 ± 1.15 mm compared to 3.96 ± 0.58 mm with central pores 1.7 mm in diameter. This nuanced understanding of scaffold microarchitecture and directionality not only enhances our grasp of bone tissue engineering principles but also paves the way for the development of tailored, clinically efficacious bone substitutes.

## 5. Conclusions

The findings of our study designate filaments of 0.50 mm as the most efficacious filament-based scaffold for fostering bone regeneration, exhibiting superior bone ingrowth and bony regenerated area when juxtaposed with Fil083 and Fil125 scaffolds. This underscores the tremendous potential of tailored filament-based designs in augmenting bone tissue engineering outcomes.

Furthermore, our investigation elucidates the pivotal role of filament directionality in filament-based microarchitectures, particularly concerning scaffold designs featuring filament distances of 0.83 mm and beyond. At these distances, the directional alignment of filaments emerges as a critical determinant affecting vertical bone ingrowth and osteoconductivity. 

In light of these findings, we advocate for the optimal distance between filaments to support and guide vertical bone ingrowth and osteoconductivity to be 0.50 mm. By delineating the intricate interplay between filament characteristics and bone regeneration outcomes, our study not only advances the understanding of filament-based scaffold design but also offers valuable insights for the development of next-generation bone substitutes with enhanced efficacy and clinical translatability.

However, to bring our results closer to a clinical setting, the focus of our future studies will involve using larger animal models and addressing critical-size defect to increase the translatability of our findings.

## Data Availability

The raw/processed data required to reproduce these findings cannot be shared at this time as the data also form part of additional ongoing studies.
